# N-Acetyl Cysteine-Mediated Improvements in Dental Restorative Material Biocompatibility

**DOI:** 10.3390/ijms232415869

**Published:** 2022-12-14

**Authors:** Takanori Matsuura, Keiji Komatsu, Takahiro Ogawa

**Affiliations:** Weintraub Center for Reconstructive Biotechnology, Division of Regenerative and Reconstructive Sciences, UCLA School of Dentistry, Los Angeles, CA 90095, USA

**Keywords:** fibroblast, composite, PMMA, reactive oxygen spices (ROS), glutathione

## Abstract

The fibroblast-rich gingival tissue is usually in contact with or adjacent to cytotoxic polymer-based dental restoration materials. The objective of this study was to determine whether the antioxidant amino acid, N-acetyl cysteine (NAC), reduces the toxicity of dental restorative materials. Human oral fibroblasts were cultured with bis-acrylic, flowable composite, bulk-fill composite, self-curing acrylic, and titanium alloy test specimens. Cellular behavior and function were analyzed on and around the materials. Impregnation of the bulk-fill composite and self-curing acrylic with NAC reduced their toxicity, improving the attachment, growth, and function of human oral fibroblasts on and around the materials. These mitigating effects were NAC dose dependent. However, NAC impregnation of the bis-acrylic and flowable composite was ineffective, with no cells attaching to nor around the materials. Although supplementing the culture medium with NAC also effectively improved fibroblast behaviors, direct impregnation of materials with NAC was more effective than supplementing the cultures. NAC-mediated improvements in fibroblast behavior were associated with reduced production of reactive oxygen species and oxidized glutathione together with increased glutathione reserves, indicating that NAC effectively directly scavenged ROS from materials and reinforced the cellular antioxidant defense system. These results establish a proof of concept of NAC-mediated improvements in biocompatibility in the selected dental restorative materials.

## 1. Introduction

Polymer- or resin-based materials are routinely used in various dental procedures, including but not limited to filling cavities, making dentures, and fabricating provisional crowns and implant provisional restorations [1,2,3,4,5,6,7,8,9,10]. All these procedures either pave the way for final restorations or represent a transitional phase of treatment important for successful restoration and clinical outcomes. These materials must therefore be fully biocompatible [9,10,11,12]. There are many different polymer-based restorative materials, including self-curing poly(methyl methacrylate) (PMMA) acrylics, bis-acrylics, and composite resins. These materials have fundamentally different compositions, i.e., self-curing acrylics are based on PMMA, whereas bis-acrylics contain acrylates and methacrylates with silicate glass filler particles. Composite materials consist of bisphenol A-glycidyl methacrylate (Bis-GMA) and urethane dimethacrylate (UDMA). Furthermore, different materials use different polymerization initiators. Unfortunately, although specific toxicities remain unquantified, there are general concerns that current polymer-based materials are all toxic to some degree, compromising biocompatibility [3,8,9,10,12,13,14,15,16,17,18,19,20,21].

However, there is a critical knowledge gap regarding material biocompatibility, clinical relevancy and impact, and the choice of material [21,22,23,24,25,26,27,28,29]. For instance, in dental implant practice, where peri-implant gingival inflammation is a clinical problem, materials are empirically chosen for implant provisional restorations with little consideration of their biological properties. In cavity fillings, provisional crowns, and implant provisional restorations, polymer-based materials are often in direct contact and/or close to the gingival tissue. For instance, provisional crowns and cavity fillings can be either sub-gingival or supra-gingival, depending on the prosthetic strategy and extent of decay, while implant provisional restorations are mostly placed sub-gingivally. Given these diverse clinical situations, the biological impact of even a single material might be quite variable.

N-acetyl cysteine (NAC), the acetylated form of the amino acid L-cysteine, is a biochemically safe molecule used in the management of various diseases such as pneumonia and bronchitis [30,31,32,33,34,35]. Its thiol (sulfhydryl) group imparts a direct antioxidant effect and neutralizes free radicals. NAC is also a precursor of glutathione, the strongest antioxidant scavenger in the body. It has been shown that adding NAC into orthopedic acrylic bone cement significantly mitigates its toxicity by effectively scavenging polymerization radicals [36,37,38,39]. Functionally, osteoblasts can survive on NAC-impregnated bone cement but not on control bone cement [38,40]. Orthopedic bone cement and dental materials based on polymers differ with respect to their other ingredients and polymerization initiators [37,41], and of the many polymer-based materials available, most have not been tested in the context of dental restorations [42,43,44,45,46,47,48]. In particular, dental restorative materials with photoinitiators are empirically considered less toxic due to their presumed complete polymerization, but this still requires biological proof.

Here, we quantified the biocompatibility of different dental restorative materials (self-curing PMMA, bis-acrylic, and two types of composite resin, with titanium alloy used as a biocompatible control) by examining their impact on the behavior and function of human oral fibroblasts. Fibroblast functions were examined both in direct contact with and in close proximity to the materials. Materials with low biocompatibility were impregnated with NAC in an effort to decrease their toxicity. Subsequently, we explored the mechanisms underlying NAC-mediated detoxification by examining intracellular reactive oxygen species (ROS) production and glutathione redox system stimulation.

## 2. Results

### 2.1. Initial Cell Attachment

**Figure 1:**
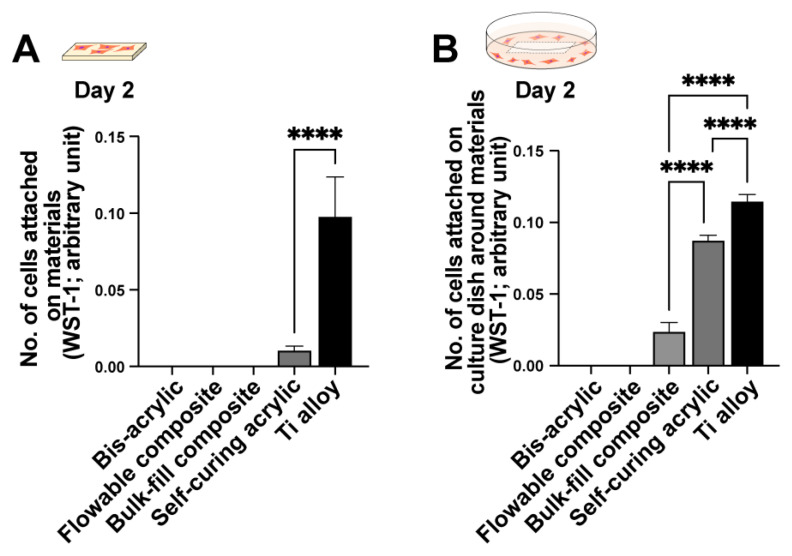
Initial attachment of fibroblasts to and around various dental restorative materials. Titanium alloy was used as a control. Contact (**A**) and proximity (**B**) experiments were performed. The WST-1 assay was performed on culture day two to quantify the number of cells attached to each material (**A**) and in the culture well around each material (**B**): **** *p* < 0.0001.

Initial cell attachment was evaluated after two days of culture in both contact (i.e., growing on the material) and proximity (i.e., growing around the material) assays. In contact experiments, fibroblasts attached to the titanium alloy and minimally to the self-curing acrylic, but not to bis-acrylic, flowable composite, or bulk-fill composite materials (Figure 1A). In proximity experiments, fibroblasts survived and attached to the culture dish around the titanium alloy and self-curing acrylic (to a greater extent than in contact experiments; Figure 1B). There was also some fibroblast attachment to the culture dish around the bulk-fill composite.

### 2.2. Cell Proliferation

**Figure 2:**
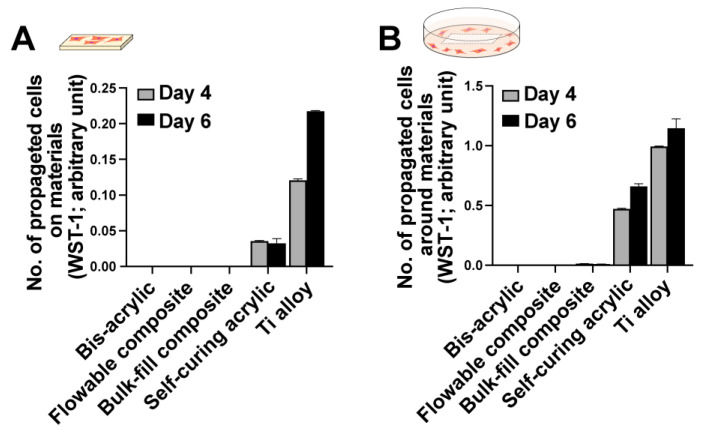
Propagation of fibroblasts on and around various dental restorative materials. Contact (**A**) and proximity (**B**) experiments were performed. The WST-1 assay was performed on culture days four and six to quantify the number of cells propagated on each material (**A**) and in the culture well around each material (**B**).

The number of fibroblasts cultured with different materials was measured after four and six days of culture in contact and proximity assays. Cells were only detected on the self-curing acrylic and titanium alloy in contact experiments (Figure 2A), increasing from day four to six on the titanium alloy but not the self-curing acrylic. In proximity experiments, more cells grew around the self-curing acrylic than in the contact experiment (Figure 2B), increasing from day four to day six. A few fibroblasts grew around the bulk-fill composite in the proximity experiment, but there was no cell growth on or around the bis-acrylic and flowable composite.

### 2.3. NAC-Dependent Improvements in Initial Cell Attachment

**Figure 3:**
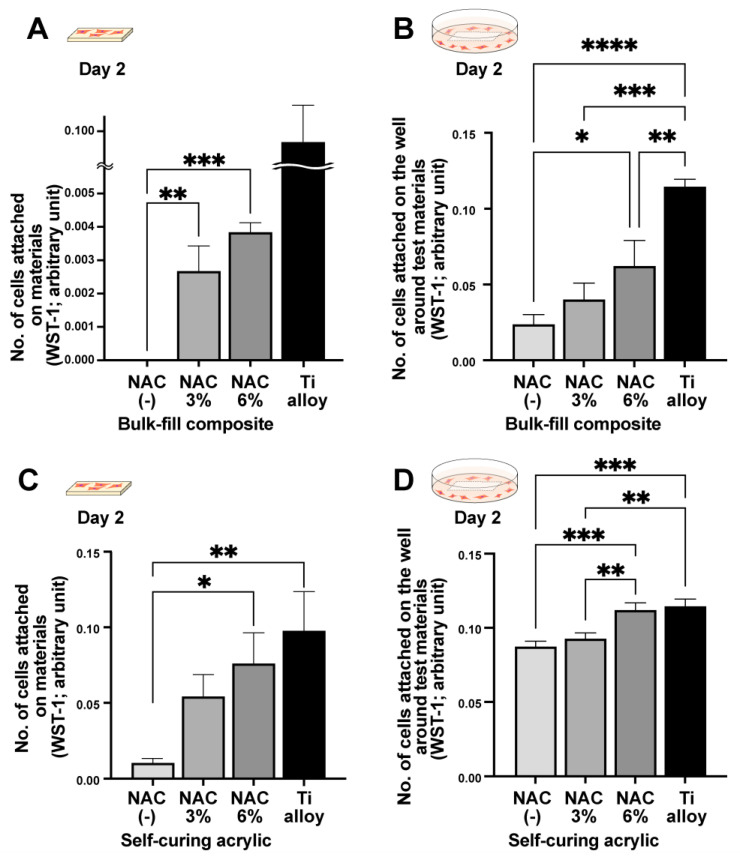
NAC-mediated improvements in material biocompatibility. NAC was impregnated into the materials at two different concentrations. Results of WST-1 assays performed on day two to quantify the number of cells attaching to bulk-fill composite (**A**) and to the well around bulk-fill composite (**B**). The number of cells attaching to self-curing acrylic (**C**) and to the well around self-curing acrylic (**D**): * *p* < 0.05, ** *p* < 0.01, *** *p* < 0.001, and **** *p* < 0.0001.

To determine whether NAC detoxifies or mitigates the toxicity of the materials, we impregnated NAC into all the materials at two different concentrations, 3% and 6%. On day two, no cells were observed on or around the bis-acrylic and flowable composite even when impregnated with NAC at either concentration. However, there was significantly more growth in and around the NAC-impregnated bulk-fill composite and self-curing acrylic than native forms (Figure 3). Some cells survived and attached on the NAC-impregnated bulk-fill composite (Figure 3A), with more cells attaching at the higher NAC concentration. In the proximity experiment, there was a dose-dependent increase in the number of cells around the NAC-impregnated bulk-fill composite (Figure 3B), while there was also a clear dose-dependent increase in the number of cells around the NAC-impregnated self-curing acrylic in both the contact and proximity experiments (Figure 3C,D). Of note, with 6% NAC, the cell count was equivalent to that observed with the titanium alloy.

### 2.4. NAC-Dependent Improvements in Cellular Propagation

**Figure 4:**
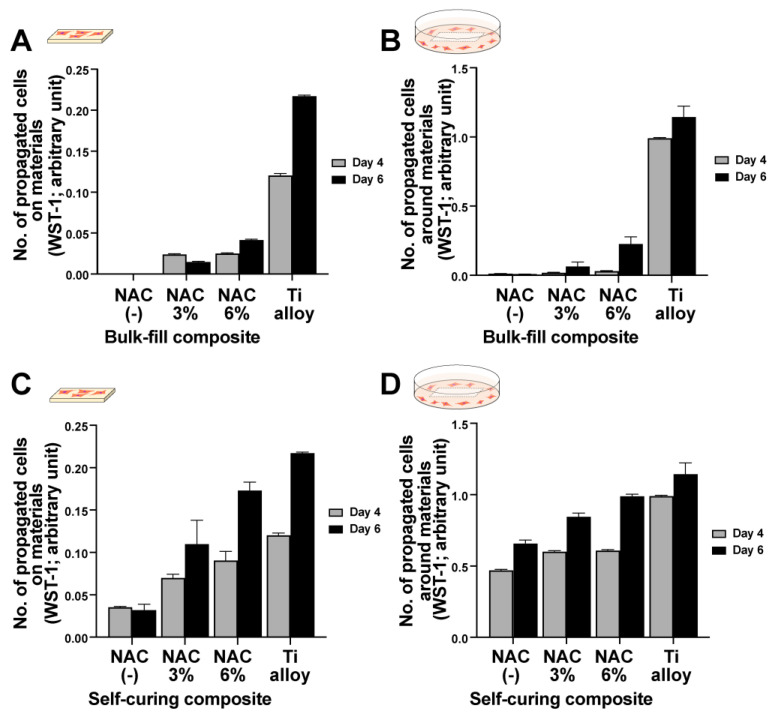
NAC-mediated improvements in material biocompatibility. WST-1 assays were performed on days four and six to quantify the number of cells proliferating on bulk-fill composite (**A**) and around bulk-fill composite (**B**). The number of cells proliferating on self-curing acrylic (**C**) and around self-curing acrylic (**D**).

**Figure 5:**
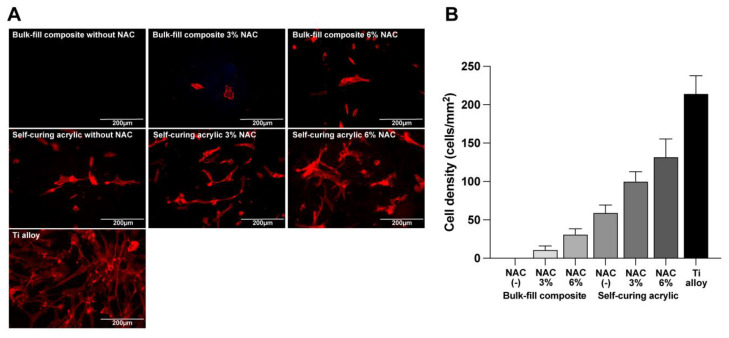
Fibroblasts visualized on materials with or without NAC impregnation. (**A**) Fluorescence microscopy images of fibroblasts on culture day four stained for cytoskeletal actin filaments (red). (**B**) Cell density was quantified based on the images.

Similar to day two cell attachment results, significantly more fibroblasts survived and propagated after NAC impregnation at four and six days of culture. No cells were quantifiable on or around the bis-acrylic and flowable composite after four and six days of culture regardless of the NAC concentration. Therefore, only the bulk-fill composite and self-curing acrylic results are presented (Figure 4A–D). In general, the number of cells increased in an NAC dose-dependent manner. NAC more effectively reduced the toxicity of the self-curing acrylic than the bulk-fill composite, with cellular proliferation on and around the self-curing acrylic similar to the titanium alloy. However, NAC had a greater relative effect on the bulk-fill composite than the self-curing acrylic, increasing proliferation from a baseline of zero. Fluorescence microscopy with image-based cell counting confirmed an NAC dose-dependent effect on the bulk-fill composite and self-curing acrylic (Figure 5).

### 2.5. NAC-Dependent Changes in Fibroblast Gene Expression

**Figure 6:**
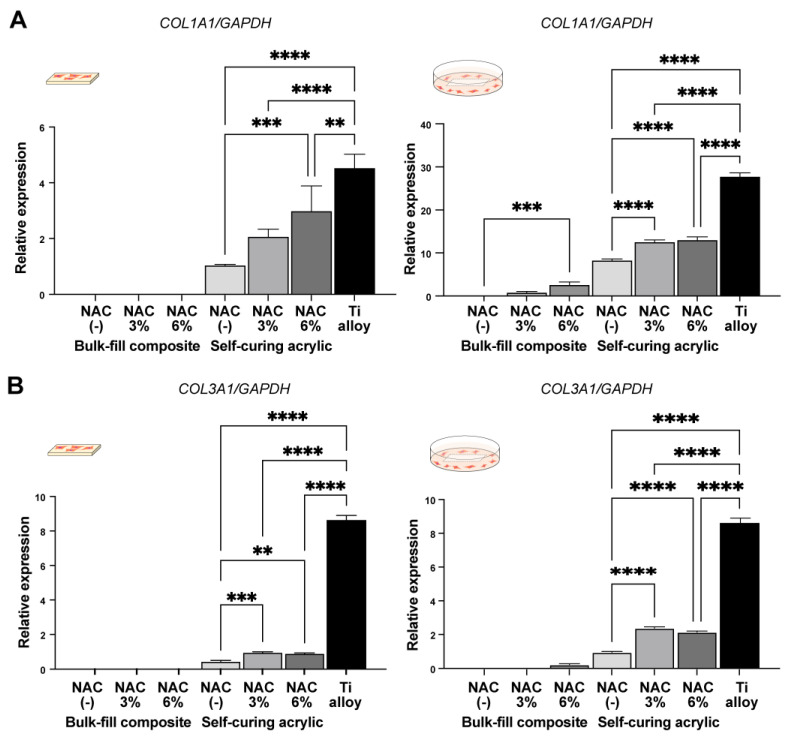
NAC-mediated improvements in material biocompatibility. Real-time PCR for collagen type 1 (**A**) and 3 (**B**) genes conducted on day four are shown. RNA was extracted from contact and proximity experiments: ** *p* < 0.01, *** *p* < 0.001, **** *p* < 0.0001.

We next examined the expression of fibroblast-related genes in fibroblasts grown on and around the materials with or without NAC. Given that no cells were detected on or around the bis-acrylic and flowable composite regardless of NAC impregnation, only results for the bulk-fill composite and self-curing acrylic are presented (Figure 6). In contact experiments, fibroblasts growing on the self-curing acrylic expressed significantly more type 1 collagen alpha in an NAC dose-dependent manner (Figure 6A). Collagen type 3 alpha expression also increased in cells grown on the self-curing acrylic impregnated with NAC, but to a much lesser degree than on the titanium alloy (Figure 6B). No collagen type 1 or 3 expression was detected in cultures with the bulk-fill composite with or without NAC in contact experiments (Figure 6A,B). In proximity experiments, there were significant increases in both collagen type 1 and 3 expression in the self-curing acrylic cultures with NAC, but not in a dose-dependent manner (Figure 6A,B). In cultures with the bulk-fill composite, the gene expression was exclusively detected in the presence of NAC impregnation.

### 2.6. Effect of NAC Administration Type on Biocompatibility

**Figure 7:**
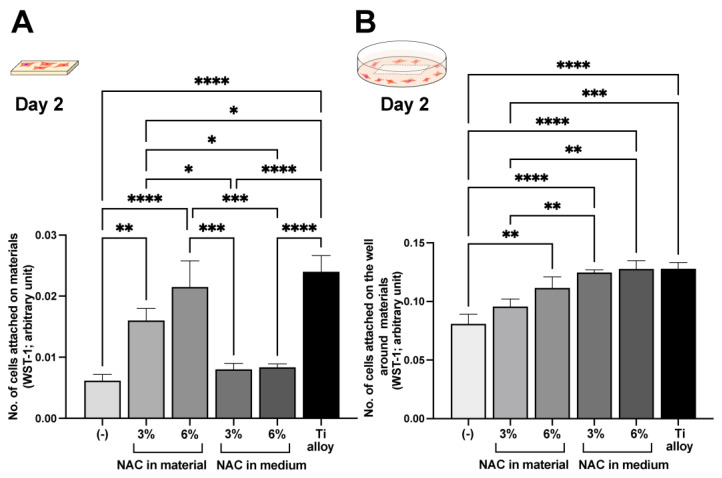
Effects of NAC administration methods. NAC was administered by material impregnation or by supplementing in the culture medium. The WST-1 assay was conducted on culture day two to quantify the number of cells attached to each material (**A**) and to the culture well around each material (**B**). Note that the same amount of NAC was added between the impregnation and supplementation methods. See the Materials and Methods sections for details: * *p* < 0.05, ** *p* < 0.01, *** *p* < 0.001, and **** *p* < 0.0001.

We next examined the effect of NAC impregnation or culture supplementation on cell counts on and around the self-curing acrylic on day two. In contact experiments, NAC impregnation more effectively promoted cell attachment than supplementation (Figure 7A); indeed, adding NAC to the medium failed to significantly increase the cell count compared with control. Conversely, in proximity experiments, supplementing the medium with NAC was more effective than impregnation (Figure 7B), with an equivalent number of cells counted around the self-curing acrylic as around the titanium alloy.

### 2.7. NAC-Dependent Activation of the Antioxidant Redox System

**Figure 8:**
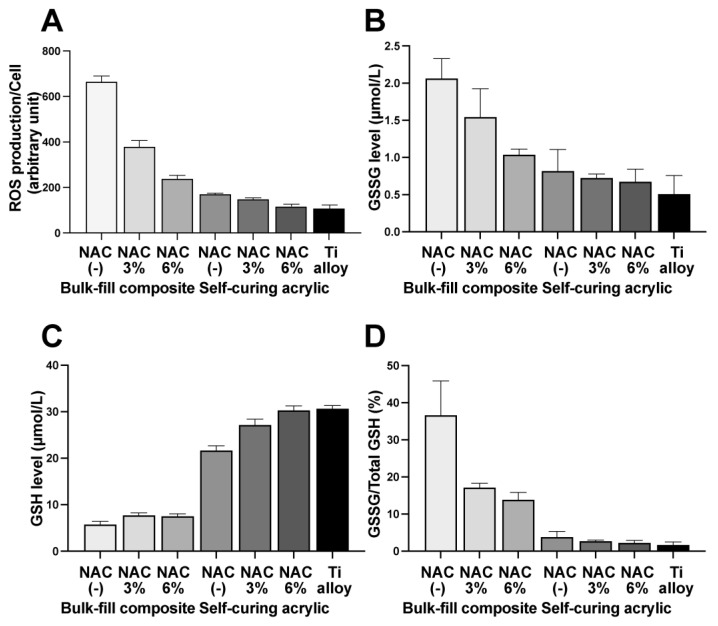
NAC-mediated improvements in antioxidant defenses. (**A**) ROS levels in fibroblasts collected from the proximity experiment. (**B**) GSSG (glutathione disulfide, an oxidized form of glutathione) levels in fibroblasts collected from proximity experiment. (**C**) GSH (a reduced form of glutathione) levels in fibroblasts collected from proximity experiments. (**D**) GSSH/GSH in fibroblasts collected from the proximity experiment.

To understand the mechanism underlying the NAC-mediated effects on fibroblast viability and gene expression, we first assessed NAC-dependent changes in reactive oxygen species (ROS) production by fibroblasts in proximity experiments around the bulk-fill composite and self-curing acrylic. There was a clear trend in decreasing ROS production with increasing NAC concentration for both materials (Figure 8A), especially for the bulk-fill composite due to the higher baseline. ROS levels around the 6% NAC-impregnated self-curing acrylic were nearly equal to those around the titanium alloy.

Finally, we examined reduced and oxidized glutathione (GSH/GSSG) levels in fibroblasts in the proximity assay (Figure 8B–D). GSSG decreased around the bulk-fill composite impregnated with NAC in a dose-dependent manner (Figure 8B), whereas GSSG levels around the self-curing acrylic remained low and were not significantly affected by NAC. Although GSH levels were low around the bulk-fill composite, NAC impregnation significantly increased the levels (Figure 8C). NAC significantly increased GSH levels around the self-curing acrylic in a dose-dependent manner, while the GSSG/GSH ratio decreased with increasing NAC around both materials (Figure 8D).

## 3. Discussion

Here we show that NAC can mitigate against the adverse cytotoxic effects of polymer-based dental restoration materials. There were, however, limits to the observed effect at the NAC concentrations tested here; for instance, NAC did not significantly improve the biocompatibility of the flowable composite to exert a biological impact. Self-curing acrylic was most improved by NAC, with impregnation with 6% NAC producing similar biocompatibility to the titanium alloy, i.e., improving the biocompatibility from biotolerant to bioinert [49,50,51,52,53,54,55]. NAC impregnation of the bulk-fill composite resulted in some cell attachment from a baseline of zero without impregnation, i.e., improving the material biocompatibility from lethal to biotolerant. Of note, the biological impact differed depending on whether the fibroblasts were in contact with or in close proximity to the materials, with a general trend of lower material toxicity in proximity experiments with fibroblast attachment and gene expression even for the bulk-fill composite. In particular, initial cell attachment in proximity to the NAC-impregnated self-curing acrylic was similar to that around the titanium alloy, albeit with subsequently impaired proliferation and gene expression at later time points.

Discriminating between the effects of NAC impregnation and supplementation provided important mechanistic insights. NAC impregnation of the material, but not the medium, significantly improved cellular attachment to self-curing resin. However, adjacent cells benefitted much more from NAC supplementation of the medium. These results indicate that (1) the material was very toxic and treatment of the cells was not sufficient to maintain cellular viability; (2) to rescue cells growing on materials, detoxification of the material rather than the environment with NAC was necessary; (3) cells adjacent to the material are subjected to less toxicity, so NAC treatment in the medium was more effective; (4) nonetheless, impregnation of the material with NAC was effective both for contacting and adjacent cells.

There are three possible pathways by which NAC might improve cellular function on and around the materials. The first is NAC-mediated scavenging of free radicals, monomer remnants, and other ROS within materials; NAC acts as a direct ROS scavenger due to its redox potential as a thiol donor [38,39,43,44,56,57]. A significant reduction in polymerization radicals and other oxidative stresses by NAC was demonstrated in bone cement [38,58]. This pathway could explain the considerable improvement in cellular function seen in both contact and proximity experiments. Indeed, levels of GSSH (glutathione disulfide), the oxidized form of glutathione, decreased with increasing NAC, indicating that glutathione (GSH), a major redox system within cells, was not depleted. The second pathway is the release of NAC from a material into the local environment, where it might permeate into or be taken up by cells. In addition to direct scavenging, NAC is a precursor of glutathione, a major antioxidant defense system, and it increases glutathione reserves. The third pathway would be NAC scavenging ROS released from a material. The latter two pathways could in part explain the biological improvements seen in proximity experiments. These two pathways were demonstrated in osteoblasts on NAC-impregnated bone cement [38]. Indeed, this study demonstrated that the ROS in fibroblasts were inversely correlated with the impregnated NAC dose. Furthermore, cellular GSH increased with NAC concentration in the material, indicating that NAC was released from the materials and exploited to increase cellular glutathione reserves. The plausibility of the second and third pathways playing a role is also supported by the positive results from the experiment in which the medium was supplemented with NAC.

For clinical correlation, it was necessary to examine both the effects of materials in direct contact with and in close proximity to cells to simulate the clinical context. The marked differences in the contact and proximity results suggest a need for separate assessments depending on the biological and clinical contexts and validate the model used in this study. Our model was also able to differentiate between different cellular responses caused by different materials. The test specimens occupied a quarter of a 12-well culture dish well. A very small specimen would be insufficient to count adherent or attaching cells and may not have a sufficient biological impact. Conversely, a large specimen might prevent any cellular growth. Other techniques exist to examine the biological effects of materials, such as the use of extracts from materials; however, the concentration and quantity of extract to use in culture are critical and difficult to optimize, and, more importantly, the contact effect cannot be evaluated. Our optimized model has the advantages of using the actual test specimens and the ability to test both contact and non-contact effects. Regarding the time points of assays, cell attachment is usually assessed within one day of culture. However, this study dealt with toxic materials, and the number of cells attached to the materials was anticipated to be very low. The result of the WST-1 assay could be none, even though there were a few cells that survived and attached, due to the value being lower than the detection limit of the assay. Further, as mentioned above, the surface area of the test specimens was only a quarter of a single well of the 12-well culture dish, which made the detection more difficult. Therefore, considering the sensitivity and validity, we evaluated the cell attachment on day 2 in this particular culture model.

The negative impact of the materials tested was more than anticipated, with no cells surviving and attaching to the bis-acrylic, flowable composite, or bulk-fill composite [24,56,59,60]. The self-curing acrylic was the most biocompatible material tested. The composite materials used a photoinitiator, and polymerization is supposed to be complete by the end of light irradiation. Of note, the composite materials were more cytotoxic than the self-curing acrylic, which is considered to take longer to polymerize [3,4,5,61]. Our results not only highlight some of the limitations of currently used dental restorative materials but also provide a potential solution in exploiting the biocompatibility effects of NAC to develop or improve future materials. In particular, the NAC-impregnated self-curing acrylic reached (for some parameters) equivalent biocompatibility to the titanium alloy, a bioinert material. Although we examined four different representative materials, other commercial and experimental materials are available that would be worth studying, and NAC concentrations could be further optimized. To date, there have been no comparative studies of the effect of NAC in several materials, but here we report material-specific NAC efficacy due to highly variable baseline biocompatibility and the behavior of polymerization, suggesting the need for material-specific NAC optimization.

## 4. Materials and Methods

### 4.1. Material Preparation and Characterization

**Figure 9:**
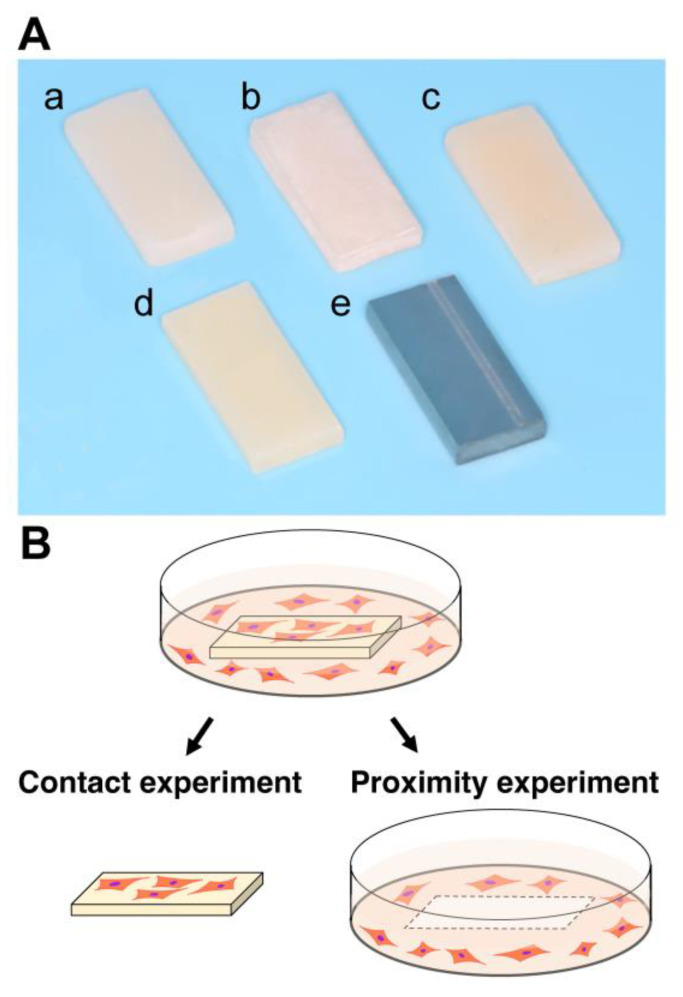
(**A**) Test specimens made of bis-acrylic (**a**), flowable composite (**b**), bulk-fill composite (**c**), self-curing acrylic (**d**), and grade 5 titanium alloy (**e**). (**B**) Culture experiment design. The contact and proximity experiments were conducted separately to differentiate the reaction of cells in direct contact with and close to each material.

Five different test materials in rectangular plate form (6 mm × 14 mm, 2 mm thickness) were prepared (Table 1, Figure 9A). Bis-acrylic, flowable composite, bulk-fill composite, and self-curing acrylic were prepared using standardized silicone molds and according to the manufacturers’ instructions. All acrylic plates were washed with a steam cleaner and disinfected with 75% ethanol. Test plates impregnated with NAC were also evaluated. NAC was prepared as a 2 M stock solution in HEPES buffer, pH 7.2–7.5. For self-curing acrylic, the NAC solution was put into the monomer first and then mixed with the polymer powders for polymerization. For composite materials, the NAC solution was spatulated into the composite and light-cured according to the manufacturer’s instructions. The final NAC concentrations impregnated in the materials were adjusted to 3% and 6% *w*/*w*. To supplement NAC in the culture media, the amount of NAC corresponding to the above-mentioned 3% and 6% *w*/*w* was added into the culture medium.

### 4.2. Cell Culture

Human gingival fibroblasts were purchased from ScienCell Research Laboratories (Carlsbad, CA, USA) and grown in a fibroblast medium supplemented with 5% FBS, 1% fibroblast growth supplement-2, and 1% penicillin/streptomycin solution. At 80% confluence, cells were detached using 0.05% trypsin-EDTA and seeded onto subsequent culture plates. Passage 5–8 cells were seeded onto each test material placed in a well (20 mm diameter) of 12-well culture plates at a density of 4 × 10^4^ cells/well. The culture medium was renewed every three days. This study was approved by the UCLA Institutional Biosafety Committee (BUA-2-22-036-001).

### 4.3. Quantification of Attached and Propagated Cells

The number of attached fibroblasts was counted in (i) contact and (ii) proximity experiments, the former being the quantification of fibroblasts attached to test materials and the latter quantification of fibroblasts attached to the wells around the materials (Figure 9B). The water-soluble tetrazolium salt (WST-1)-based colorimetric assay was used to quantify the cell number. Attached fibroblasts were measured two days after seeding, while propagated fibroblasts were measured four and six days after seeding. The amount of formazan product was measured at 450 nm using a microplate reader (Synergy H1 microplate reader, BioTek Instruments, Winooski, VT, USA).

### 4.4. Cell Visualization

Cells were visualized and observed using fluorescence microscopy (DMI6000B, Leica Microsystems, Wetzlar, Germany) four days after seeding. Fibroblasts were stained with rhodamine-phalloidin for actin filaments. The density of fibroblasts on each test material was obtained by counting cells in the images.

### 4.5. Gene Expression Analysis

Total RNA was extracted from cells on day four attached to materials and the wells around the materials using TRIzol reagent (Life Technologies, Carlsbad, CA, USA) and purified using the Direct-zol™ RNA MiniPrep kit (Zymo Research, Irvine, CA, USA) according to the manufacturers’ protocols. RNA was quantified with a NanoDrop™ One (Thermo Fisher Scientific, Waltham, MA, USA). Reverse transcription of total RNA was performed with a SuperScript^®^ VILO ™ cDNA Synthesis Kit (Invitrogen, Carlsbad, CA, USA). Real-time quantitative polymerase chain reaction (PCR) was performed with the QuantStudio^TM^ 3 Real-Time PCR System (Applied Biosystems, Waltham, MA, USA) to quantify expression of collagen type I alpha 1 (*Col1a1*; assay ID: Hs00164004_m1, Applied Biosystems) and collagen type III alpha 1 (*Col3a1*; assay ID: Hs00943809_m1, Applied Biosystems) mRNA. Gene expression levels were normalized to glyceraldehyde-3-phosphate dehydrogenase (*Gapdh*; Assay ID: Hs02786624_g1, Applied Biosystems) expression.

### 4.6. Intracellular ROS Detection

Intracellular ROS were detected using the OxiSelect™ Intracellular ROS Assay Kit (Cell Biolabs, Inc., San Diego, CA, USA). Cells cultured for two days were incubated and loaded with 1 mM 2′, 7′-dichlorodihydrofluorescin diacetate (DCFH-DA) for 60 min at 37 °C. Each test material was then placed in each well. Afterwards, excessive DCFH-DA and test materials were removed and washed with PBS. Oxidized DCF was measured using a microplate reader at excitation and emission wavelengths of 480 and 530 nm, respectively.

### 4.7. Intracellular Glutathione Detection

To quantify total intracellular glutathione, a 5,50-dithiobis(2-nitrobenzoic acid) (DTNB)-based total glutathione quantification kit (Dojindo Molecular Technologies, Inc., Gaithersburg, MD, USA) was used. Oxidized glutathione (GSSG) was detected by masking reduced glutathione (GSH) with a masking reagent. Three days after seeding on test materials, the cells around materials were collected and lysed, and the supernatant was incubated with DTNB and glutathione reductase for 10 min at 37 °C. Total glutathione and GSSG concentrations were determined by measuring the absorbance at 405 nm.

### 4.8. Statistical Analysis

The results are expressed as means ± standard deviations (SD). Comparisons between multiple groups, such as between 0% NAC, 3% NAC, and 6% NAC, were performed with one-way analysis of variance (ANOVA) followed by the Tukey–Kramer post-hoc test. Comparisons of two groups were performed using the unpaired Student’s *t*-test; *p*-values < 0.05 were statistically significant.

## 5. Conclusions

NAC impregnation of bulk-fill composite and self-curing acrylic reduced their toxicity and improved the behavior and function of human oral fibroblasts on and around the materials. The mitigating effects were NAC dose dependent. However, NAC impregnation was not effective for the bis-acrylic and flowable composite. Although supplementing the culture medium with NAC also improved fibroblast behavior, direct NAC impregnation into the materials was more effective. The NAC-mediated improvements in fibroblastic behavior and function were corroborated by an observed reduction in cellular ROS and an increase in cellular glutathione, indicating that NAC effectively scavenged ROS within materials and reinforced the cellular antioxidant defense system. These results establish a proof of concept of NAC-mediated improvements in biocompatibility of dental restorative materials.

## Figures and Tables

**Table 1:** Materials used in this study.

Material	Principal Ingredient
Bis-acrylic (Integrity^®^ Multi-Cure Temporary Crown and Bridge Material, Dentsply Sirona Inc., Charlotte, NC, USA)	Acrylates and methacrylates (bis- and multifunctional) Barium boro alumino silicate glass
Flowable composite (Aelite™ Flo, BISCO Inc., Schaumburg, IL, USA)	Bis-GMA
Bulk-fill composite (Aelite™ Aesthetic Enamel, BISCO Inc.)	Bis-GMA, UDMA
Self-curing acrylic (UNIFAST™ Trad, GC corporation, Tokyo, Japan)	Liquid: MMA Powder: PMMA
Ti alloy	Ti-6Al-4V

Abbreviations: Bis-GMA, bisphenol A glycidyl methacrylate; UDMA, urethane dimethacrylate; MMA, methyl methacrylate; PMMA, poly(methyl methacrylate).

## Data Availability

The data presented in this study are available on request from the corresponding author.
